# Distinct Effects of Anxiety and Depression on Updating Emotional Information in Working Memory

**DOI:** 10.3390/ijerph20010544

**Published:** 2022-12-29

**Authors:** Yuting Zhang, Teresa Boemo, Zhiling Qiao, Yafei Tan, Xu Li

**Affiliations:** 1 Key Laboratory of Adolescent Cyberpsychology and Behavior (CCNU), Ministry of Education, Wuhan 430070, China; 2 School of Psychology, Central China Normal University, Wuhan 430079, China; 3 Key Laboratory of Human Development and Mental Health of Hubei Province, Wuhan 430079, China; 4 Faculty of Psychology, Complutense University of Madrid, 28040 Madrid, Spain; 5 Department of Neuroscience, Research Group Psychiatry, Center for Clinical Psychiatry, KU Leuven, 3000 Leuven, Belgium

**Keywords:** anxiety, depression, emotional valence, working memory updating

## Abstract

Anxiety and depression have been shown to negatively influence the processing of emotional information in working memory. However, most studies have examined anxiety-related or depression-related working memory deficits independently, without considering their high co-morbidity. We tested the effects of emotional valence on working memory performance among healthy young adults with varying levels of anxiety and depressive symptoms. Ninety young adults aged between 18–24 (51 female) completed an emotional 2-back task in which positive, negative, and neutral images were presented. Multi-level modeling was used to examine anxiety and depressive symptoms as predictors of response accuracy and latency across the three emotional valence conditions. The results showed that participants responded to negative images with the highest accuracy and to positive images with the lowest accuracy. Both negative and positive images elicited slower responses than neutral images. Importantly, we found that more severe anxiety symptoms predicted a smaller difference in response accuracy between negative and neutral stimuli, whereas more severe depressive symptoms predicted a larger updating reaction time difference between positive and neutral stimuli. These findings demonstrated the uniquely anxiety-related deficits in processing negative contents and the uniquely depression-related deficits in updating positive contents in working memory, thus highlighting the necessity of novel cognitive bias modification interventions targeting the anxiety-specific and depression-specific deficits in working memory.

## 1. Introduction

Working memory (WM) refers to the ability to actively retain and manipulate the information that is most required in ongoing tasks over a brief period of time [1,2]. WM has a central role in cognition and has been shown to be a significant predictor of academic achievement [3], language comprehension [4], and other higher-order cognitive functions [5]. Given the limited capacity of WM, researchers have increasingly focused on identifying individual characteristics that might have an impact on WM performance.

Previous evidence has shown that anxiety and depressive symptoms can impair WM. According to the attentional control theory [6], anxiety reduces the processing and storage capacity of WM, thus producing detrimental effects on WM performance. Consistent with this assumption, a meta-analysis of 177 studies on the association between self-reported anxiety and WM performance showed that anxiety severity was negatively related to WM across WM tasks, differing only in effect size magnitude [7]. In the case of depression, cognitive dysfunctions are described as a core diagnostic and symptomatic feature of patients suffering from this condition [8], and impairments in WM have been found to be a key vulnerability factor for depression [9,10,11]. Indeed, the results of several studies have shown that patients with clinical depression perform more poorly than healthy controls on various WM tasks [12,13,14]. Reduced WM capacity and poor ability to resist task-irrelevant distractors within WM have also been reported among individuals with dysphoria [15]. Furthermore, a recent meta-analysis suggested that depression-related impairments in WM persist into the remission period [16].

The picture of the effects of anxiety and depression on WM becomes more complicated when emotional materials are included in WM tasks. For instance, early attentional bias towards negative information, and difficulties in disengaging from negative stimuli in WM, have been reported in anxiety [17,18] and depression [19,20,21]. However, the results of other studies have not shown this pattern. For example, one study found that individuals with social anxiety showed *better*, not worse, WM capacity for threat words relative to neutral words [22], and in other studies, there was no difference between individuals with social anxiety and controls in resisting negative distractors within WM [23,24]. In addition, depression was found to be associated with deficits in the processing of positive information in WM [20,25], and others found that patients with depression performed at the same level as healthy controls in emotional WM tasks [26,27].

Furthermore, most research in this area has focused on the impact of either anxiety or depression on WM, without considering the considerable overlap in symptoms [28] or their high co-morbidity [29]. Studies in clinical populations have shown that the comorbidity of anxiety and depression predicts poorer outcomes than either disorder considered individually [30,31]. Deficits in WM that exist in both anxiety and depression might represent a transdiagnostic risk factor related to both conditions. However, there have been mixed results with respect to whether there is a shared deficit. Using a modified Sternberg task, Yoon et al. [24] observed depression-related deficits in inhibiting irrelevant emotional materials, both positive and negative; no such effect was found for anxiety, suggesting a depression-specific deficit in updating emotional contents in WM. However, a study of memory control in healthy adolescents observed a reverse pattern: anxiety was associated with difficulties forgetting negative words, whereas depression was not [32].

Taken together, the effects of emotional valence, anxiety, and depressive symptoms on WM performance are complex and inconclusive. To move forward, it is necessary to take their interactions and potentially shared and unique effects into consideration. Against this background, the current study would simultaneously investigate whether and how varying levels of anxiety and depressive symptoms affect both positive and negative WM processing. This would allow us to disentangle the debates by revealing the common and unique associations between anxiety symptoms, depressive symptoms, and emotion WM. In addition, this study would focus on a non-clinical sample of young adults. Early adulthood is a period during which many psychiatric disorders could emerge. For example, a recent meta-analysis of 192 worldwide epidemiological studies found that the proportion of individuals with onset of anxiety/fear-related disorders and mood disorders before the age of 25 were 73.3% and 34.5%, respectively [33]. Thus, examining subclinical symptoms among non-clinical young adults could provide valuable information for identifying risk markers of psychopathology [34]. Furthermore, we would take the methodology into consideration, using the multi-level modeling (MLM) technique to characterize the various levels of variation in data. Most of the previous literature used repeated measures analysis of variance (ANOVA) or analysis of covariance (ANCOVA) that focused on group means. This is problematic, as information about the response patterns within individuals has been ignored. In contrast, rather than aggregating performance across trials to form individual-by-condition scores, MLM takes both trial-by-trial variation and variation across individuals into account [35,36], thus it is more informative.

In sum, the current study aims to examine the extent to which the processing of emotional information in WM is altered with increasing severity of anxiety and depressive symptoms in young adults. To this end, we administered an emotional 2-back task in which positive, negative, and neutral images were presented. Based on previous studies [21,37,38], we hypothesized that positive and negative stimuli would be associated with higher accuracy but slower response than neutral stimuli. Given the negative associations between anxiety or depression and WM [7,20], we hypothesized that more severe anxiety or depressive symptoms would be related to poor WM performance, especially when negative contents were updated. In addition, as multiple studies have suggested an association between depression and positive attenuation [20,39,40], we hypothesized that the severity of depressive symptoms would be also related to difficulties in updating positive contents in WM.

## 2. Materials and Methods

### 2.1. Participants

Ninety-five young adults were recruited from a university in Wuhan, China via online advertising. The sample size of about 100 participants was informed by a recent study that tested the association between math anxiety and WM updating with the MLM approach [41]. Participants were excluded when they had a self-reported history of brain injury, neurological or psychiatric disorder, and data from three participants who were diagnosed with major depressive disorder were not analyzed. The remaining sample included 92 participants (56.5% female) aged between 18 to 24 (*M* = 20.46, *SD* = 1.33). This study was approved by the Ethics Committee of the university with which the first author is affiliated (approval code: CCNU-IRB-20200901). Written informed consent was obtained from all participants at the beginning of the study in accordance with the Declaration of Helsinki.

### 2.2. Self-Report Measures

#### 2.2.1. Symptoms of Anxiety

We used the State-Trait Anxiety Inventory-Trait Scale (STAI-T) [42,43] to measure symptoms of anxiety in our sample. This scale has been developed to measure one’s predisposition to anxiety and widely used in previous studies [44,45]. It contained 20 items, with each item scoring from 1 to 4. Higher scores indicated higher levels of trait anxiety.

#### 2.2.2. Symptoms of Depression

Depressive symptoms were measured with the Beck Depression Inventory-II (BDI-II) [46,47], which contained 21 items. Each item was scored from 0 to 3 and higher scores indicated more severe depressive symptomology.

### 2.3. Materials and Apparatus

Emotional images were selected from the International Affective Picture System (IAPS) [48]. In a pilot study, 111 images (37 positive, 37 negative, and 37 neutral) were evaluated by 32 undergraduate students aged between 18 and 23 (68.8% female, *M*_age_ = 19.94, *SD*_age_ = 1.39) who did not participate in the formal experiment. The students were asked to rate each image regarding valence and arousal on a 9-point Likert scale, in which 1 represented “extremely unpleasant” or “extremely calm” and 9 represents “extremely pleasant” or “extremely excited.” The positive, neutral, and negative images had mean valence rating of 6.90 (*SD* = 0.30), 4.99 (*SD* = 0.26), and 2.60 (*SD* = 0.58), with mean arousal ratings of 5.84 (*SD* = 0.70), 3.70 (*SD* = 0.34), and 5.62 (*SD* = 0.51), respectively. Consistent with a previous study [49], positive and negative images were rated higher in arousal than neutral images.

Emotional WM updating function was assessed with the emotional 2-back task (programmed in E-Prime 2.0). Participants were instructed to indicate whether the emotional valence (positive, negative, neutral) of the current image (trial *n*) was the same as the image appeared two trials earlier (trial *n*-2), and they were asked to press “F” if these two images had the same valence (match trial) or press “J” if these two images differed on valence (non-match trial). The number of match trials and non-match trials was the same. Each block started with a fixation lasting for 500 ms, and then the IAPS image appeared on the screen for 2000 ms, followed by a 2500 ms interval. Participants had a response window of 4000 ms and were asked to respond as soon as possible. The task included 330 trials and was divided into 6 blocks of 55 trials each. The first two trials in each block were not analyzed, resulting in 318 valid trials in total. WM updating performance was measured with response accuracy (proportion of correct trials) and the reaction time (RT) of correct trials.

### 2.4. Data Analysis

#### 2.4.1. Data Preparation

Trials with RT less than 200 ms were removed as outliers before analysis [50]. Participants were to be excluded if they had scores that were 3 *SD* higher or lower from the group mean in accuracy or RT; two participants were excluded due to their very low accuracy. The final sample consisted of 90 individuals (51 female), with accuracy ranging from 0.58 to 0.95 (*M* = 0.81, *SD* = 0.08).

#### 2.4.2. Statistical Analysis

Analyses were conducted using two-level MLMs, with the lmer4 [51] and lmerTest [52] package in RStudio 2021.09.1 [53]. The emotional valence condition (Level 1) nested within individuals (Level 2). Because the emotional valence condition was a categorical variable, a dummy variable was made with neutral condition designated as the reference category (D0), with D1 representing the contrast between positive and neutral conditions, and with D2 representing the contrast between negative and neutral conditions. To examine the effect of anxiety and depressive symptoms on WM updating performance, MLM analyses were conducted, with response accuracy and RT as the dependent variable, respectively. Because response accuracy is a binary score, a generalized linear mixed model (GLMM) was used and fitted with maximum likelihood (Laplace Approximation) estimation; for models with RT as the outcome, the MLM was fitted with restricted maximum likelihood (REML) estimation. Independent variables were emotional valence, the STAI-T scores, the BDI-II scores, the interaction between the STAI-T scores and emotional valence, and the interaction between the BDI-II scores and emotional valence. Separate analyses were also conducted to offer a comparison between the current and previous studies. That is, anxiety and depressive symptoms were tested separately as predictors of emotional WM performance. However, since our main aims were to take the co-occurrence of anxiety and depressive symptoms into consideration, we chose not to interpret these separate models. Nevertheless, the results were still reported for interested readers. Participant age and gender were entered into all the models as covariates.

## 3. Results

### 3.1. Descriptive Results

Participants’ STAI-T scores ranged from 28 to 62 (*M* = 42.98, *SD* = 8.53), with a Cronbach’s α value of 0.86, and their BDI-II scores ranged from 0 to 30 (*M* = 9.74, *SD* = 7.70), with a Cronbach’s α value of 0.89.

Scores on the two measures were highly correlated (*r* = 0.697, *p* < 0.001), supporting the relatively high co-occurrence of anxiety and depressive symptoms in non-clinical populations. However, this correlation coefficient was lower than the threshold of 0.8 for measuring multicollinearity [54], indicating that any collinearity would be unlikely to bias the results of further analyses. Descriptive statistics for behavioral performance on the 2-back task are provided in Table 1.

### 3.2. WM Updating Accuracy

When anxiety, depression, and each variable’s interaction with emotional valence were entered into the model (Table 2, Model 1), we found a significant main effect of emotional valence on accuracy: positive stimuli were updated less accurately than neutral stimuli (D1), *β* = −0.23, *p* < 0.001, while negative stimuli were updated at a higher accuracy than neutral stimuli (D2), *β* = 0.40, *p* < 0.001. There was no significant main effect of either anxiety or depression. A significant interaction effect between anxiety and D2 was found, *β* = −0.02, *p* = 0.011, suggesting an attenuation effect of anxiety on the updating accuracy difference between negative and neutral stimuli. There was no significant interaction effect of anxiety × D1, depression × D1, or depression × D2.

When only anxiety and its interaction with emotional valence were entered (Table 2, Model 2), the significant main effect of emotional valence remained unchanged. Similarly, we did not find a significant main effect of anxiety, *p* = 0.679. The interaction effect between anxiety and D2 was still significant, *β* = −0.02, *p* = 0.005. Additionally, we found a significant interaction effect between anxiety and D1, *β* = −0.01, *p* = 0.021; that is, anxiety attenuated the updating accuracy difference between positive and neutral stimuli.

When only depression and its interaction with emotional valence were considered (Table 2, Model 3), the significant main effect of emotional valance still remained (*p* < 0.001, *p* < 0.001). The main effect of depression was not significant, *p* = 0.727. A significant interaction was found between depression and D1, *β* = −0.01, *p* = 0.028: more severe depressive symptoms were associated with a smaller difference in updating accuracy between positive and neutral stimuli. No significant interaction effect was detected between depression and D2, *p* = 0.154.

### 3.3. WM Updating RT

When anxiety, depression, and each variable’s interaction with emotional valence were entered into the model (Table 3, Model 1), we found a significant main effect of emotional valence: positive and negative stimuli elicited slower responses than neutral stimuli, *β* = 21.91, *p* = 0.015; *β* = 30.84, *p* = 0.006. There was no significant main effect of symptoms for either anxiety or depression. A significant depression × D1 interaction on RT was found, *β* = 3.32, *p* = 0.042: more severe depressive symptoms were associated with larger updating RT differences between positive and neutral stimuli. There was no other significant interaction effect.

When only anxiety and its interaction with emotional valence were considered (Table 3, Model 2), the significant main effect of emotional valence remained; no other significant main or interaction effect was detected.

When only depression and its interaction with emotional valence were considered, the significant main effect of emotional valence, and the interaction effect between depression and D1 remained (Table 3, Model 3). Neither the main effect of depression nor the interaction effect between depression and D2 was significant.

## 4. Discussion

By undertaking a transdiagnostic approach, the current study explored the effect of anxiety and depressive symptoms on emotional WM updating performance in a non-clinical sample of young adults. Our results showed an effect of emotional valence on WM. That is, while negative stimuli were updated with highest accuracy, lowest accuracy was found for positive stimuli. Moreover, both negative and positive stimuli elicited prolonged responses relative to neutral stimuli. More importantly, more severe anxiety symptoms were found to attenuate the updating accuracy difference between negative and neutral stimuli, whereas more severe depressive symptoms were associated with larger updating RT differences between positive and neutral stimuli. These findings support domain-specific WM deficits as vulnerabilities for specific disorders.

Regarding the effects of emotional valence, negative stimuli were found to be updated with the highest accuracy, indicating the facilitation effect of negative information on WM updating. This finding is consistent with a large body of previous literature suggesting the enhanced processing of negative information within WM [55,56,57,58,59]. For example, in research using emotional facial expressions as experimental stimuli, participants showed a higher WM capacity for angry and fearful faces than for happy or neutral faces [55,59]. Furthermore, we found prolonged response latencies of negative stimuli relative to neutral stimuli. Previous electrophysiological studies have shown a larger P3 amplitude elicited by the updating of negative vs. neutral contents in WM [56,60]. The P3 amplitude is considered as an indicator of cognitive effort. Thus, the enlarged P3 indicated that more cognitive resources were allocated to maintaining and manipulating negative information than neutral information. Together, these findings suggested a biased processing towards negative information in WM. Prolonged latencies were also found in response to positive stimuli relative to neutral stimuli. We cautiously consider that, even though emotionally salient stimuli may have priority over neutral stimuli in capturing attention, extra time might be required to identify and evaluate the affective meaning of emotional images, thus resulting in the generally prolonged response latencies of emotional stimuli (i.e., both negative and positive stimuli). Additionally, we found the response accuracy was lower for positive images than for neutral and negative images, which might be related to the relatively lower adaptive value of positive information compared to negative information [61].

Importantly, we found a modulation effect of anxiety on WM. That is, more severe anxiety attenuated the differences in response accuracy between negative and neutral stimuli. It is likely that the results were driven by the detrimental effect of anxiety on the WM processing of negative information. Indeed, several studies have shown that the severity of anxiety symptoms was distinctly associated with difficulties in filtering out threat-related distractors, but not neutral distractors [17,62,63,64]. In the n-back task, information is displayed in quick succession, and participants are required to continuously replace their old WM representations with new representations, involving active attentional disengagement and shifts [65]. While negative information has priority in capturing attention, anxiety may impair this process by allocating resources to process the negative stimuli [66,67] rather than to execute the task. Consequently, few cognitive resources are available for the active monitoring and removal of representations in WM, resulting in poorer performance. These results are corroborated by the results of neuroimaging studies. Anxiety has been shown to be associated with greater activation in the amygdala in response to fearful faces than neutral faces, showing an anxiety-related hyper-responsiveness to negative information in early cognitive processing [68]. Furthermore, there is evidence for an association between anxiety symptoms and prefrontal cortex dysfunctions [69,70]. Additional research is needed to validate a process in which anxiety impairs the processing of negative information in WM by altering amygdala-prefrontal circuitry.

Furthermore, we found that more severe depressive symptoms were associated with larger updating RT differences between positive and neutral images. The main effect of valence showed that positive images elicited a longer RT than neutral images. Together, these results suggest a role of depression in slowing down the processing of positive information within WM. Our findings are consistent with the pattern of blunted positivity found in previous research. For example, studies in adolescents and young adults with subclinical depressive symptoms pointed to the link between depression and blunted positivity in WM processing [40,71]. Similar findings were also reported in patients with clinical and remitted depression [72,73,74]. The absence of preference for positive information in depression is further supported by findings from eye-tracking research. Several studies have shown that, compared to healthy controls, participants who were depressed showed lower orienting to and less gaze maintenance on positive stimuli during the early attentional process [19,75,76]. The decreased attention allocation towards positive stimuli might make it difficult for them to integrate positive stimuli into WM, thus resulting in delays in updating. A recent study added one more piece of evidence. Depressive symptoms were associated with longer response times in a digit-parity task when digits appeared near positive stimuli rather than near neutral stimuli [76]. The positive attenuation hypothesis has proposed that depression is associated with lower levels of positive emotion, reduced reactivity to positive emotional stimuli, and reduced positivity in imagining the future [77,78,79]. This perspective highlights blunted positivity as a characteristic of depression. Preference for positive information has been interpreted as a protective bias that motivates people to pursue rewarding goals and activities. There is also accumulating evidence that the active maintenance of positive experiences is a critical factor in translating hedonic experiences of reward into goal-directed actions [80]. The lack of positivity in WM might explain why anhedonia is considered a core symptom of depression [81].

Contrary to our hypothesis, we did not find an association between depression severity and the updating of negative contents in WM. This is also inconsistent with earlier literature [9,20,82]. For example, individuals with subclinical depression have been found to be slower in updating negative self-related words than individuals without depression, and the prolonged RT in updating negative words was associated with more severe rumination and depressive symptoms [82]. The discrepancy between our results and the previous findings may be due to the differences in samples. We assessed depressive symptoms on a continuum in a healthy population, whereas other studies used a clinical analogue sample of young adults who reported moderate to severe depressive symptoms [82] or a clinical sample [20]. More importantly, these earlier studies did not take the co-occurrence of anxiety into account. Studies have shown that taking co-morbidity into account can substantially change results. Researchers in several studies reported that a significant association between depression and attentional bias became nonsignificant after adjusting for anxiety [73,76]. In another study, attentional biases to negative information were found in individuals who reported high levels of depression and anxiety, but not in individuals who reported high levels of depression but low levels of anxiety [83]. In the current research in a sample of healthy young adults, deficits in updating negative contents within WM appeared to be a relatively specific vulnerability factor for anxiety rather than depression.

Our findings provide further evidence that supports the theoretical framework of cognitive models of anxiety and depression, which points out the deficits in controlling the contents of WM as one of the key cognitive vulnerability factors [9,84]. The unique effect of anxiety and depressive symptoms on specific emotional WM processes found in our study indicates disorder-specific deficits in WM processing, thus highlighting the importance of considering these unique cognitive vulnerabilities when tracking future risk for the onset and maintenance of such disorders. Furthermore, our findings have practical implications for developing interventional strategies. Current cognitive bias modification (CBM) programs aim to modify negative cognitive biases, with the distal goal of reducing subclinical or clinical symptoms of anxiety and depression. However, a recent network meta-analysis of 85 randomized control trials of CBM interventions for adults found that the CBM-Interpretation programs were more effective than control conditions in reducing symptoms of anxiety disorders, but no reliable effects were found for depressive disorders [85]. This might be due to uniquely anxiety-related deficits in the processing negative contents, while this is not the case for depression, as demonstrated in our study. Thus, developing disorder-targeted rather than universal interventions would, to a higher degree, contribute to the intervention and treatment of specific symptoms. For example, there is already limited but promising evidence of the effectiveness of interventions, including a positive imagery-based CBM [86] and brief positive episodic simulation [87], focusing on improving positive information processing to reduce depression.

The present study has several limitations. First, our samples were healthy young adults, thus the results could not be generalized to clinical samples or other age groups. Second, the present study used IAPS images that depicted emotional scenes to evoke general negative or positive emotions rather than particular negative emotions such as threat or sadness. Further research could examine whether anxiety and depression affect WM processing ascribed to some specific emotional experiences [88]. Third, the association between anxiety, depression, and WM performance might vary by cognitive load. For example, Huang et al. [89] reported depression-related deficits in the processing of negative information in WM under higher WM load rather than lower WM load. Future research could investigate whether imposing WM load strengthens or attenuates the associations between anxiety, depression, and emotional WM. Finally, we focused on how anxiety and depression are related to general updating performance, and it will be useful also to study how anxiety and depression impact specific WM updating processes, such as removal.

## 5. Conclusions

Despite the considerable conceptual overlap and co-occurrence of anxiety and depression, little is known about whether they affect the processing of emotional stimuli in WM differently. Our results showed that anxiety symptoms were uniquely associated with the impairments in updating negative information in WM, whereas depressive symptoms were uniquely associated with the impairments in the processing of positive information in WM. Therefore, the current study challenges the general conceptualization regarding the transdiagnostic role of WM impairment in anxiety and depression and elucidates the specific role of WM abnormalities associated with increased levels of anxiety or depressive symptoms. Our findings implicate the necessity for new CBM interventions designed to reduce anxiety-specific and depression-specific deficits in the processing of emotional information in WM.

## Figures and Tables

**Table 1:** Mean response accuracy and RT in different conditions.

Condition	Accuracy (%) M (SD)	RT (ms) M (SD)
Positive stimuli	75.56 (9.10)	1411 (244)
Negative stimuli	85.30 (8.99)	1419 (235)
Neutral stimuli	80.26 (9.29)	1387 (238)

Note. RT: reaction time.

**Table 2:** Results of GLMMs with anxiety and depressive symptoms as predictors of WM updating accuracy.

	Model 1			Model 2			Model 3		
	*Β* (*SE*)	*z*	*p*	*Β* (*SE*)	*z*	*p*	*Β* (*SE*)	*z*	*p*
Intercept	1.39 (0.08)	16.68	<0.001	1.39 (0.08)	16.61	<0.001	1.39 (0.08)	16.48	<0.001
Age	−0.01 (0.04)	−0.32	0.752	−0.02 (0.04)	−0.46	0.646	−0.02 (0.04)	−0.55	0.585
Gender (female)	0.13 (0.11)	1.20	0.231	0.14 (0.11)	1.29	0.199	0.14 (0.11)	1.32	0.187
D1 (positive vs. neutral)	−0.23 (0.04)	−5.62	<0.001	−0.23 (0.04)	−5.59	<0.001	−0.23 (0.04)	−5.53	<0.001
D2 (negative vs. neutral)	0.4 (0.06)	7.22	<0.001	0.4 (0.06)	7.21	<0.001	0.4 (0.06)	7.01	<0.001
Anxiety	−0.01 (0.01)	−0.93	0.351	−0.003 (0.01)	−0.41	0.679	-	-	-
Anxiety × D1	−0.01 (0.01)	−1.04	0.300	−0.01 (0.05)	−2.31	0.021	-	-	-
Anxiety × D2	−0.02 (0.01)	−2.55	0.011	−0.02 (0.01)	−2.82	0.005	-	-	-
Depression	0.01 (0.01)	0.91	0.363	-	-	-	0.003 (0.01)	0.35	0.727
Depression × D1	−0.01 (0.01)	−0.86	0.391	-	-	-	−0.01 (0.01)	−2.20	0.028
Depression × D2	0.01 (0.01)	0.76	0.447	-	-	-	−0.01 (0.01)	−1.43	0.154

Note. D1 represents the contrast between conditions with positive and neutral stimuli; D2 represents the contrast between conditions with negative and neutral stimuli. SE: standard error.

**Table 3:** Results of MLMs with anxiety and depressive symptoms as predictors of WM updating RT.

	Model 1			Model 2			Model 3		
	*Β* (*SE*)	*z*	*p*	*Β* (*SE*)	*z*	*p*	*Β* (*SE*)	*z*	*p*
Intercept	1409.1 (38.55)	36.55	<0.001	1408.82 (38.28)	36.81	<0.001	1409.54 (38.38)	36.73	<0.001
Age	−11.78 (19.80)	−0.60	0.554	−12.05 (19.42)	−0.62	0.537	−9.77 (19.49)	−0.50	0.617
Gender (female)	−36.67 (50.67)	−0.72	0.471	−36.34 (50.22)	−0.72	0.471	−37.44 (50.48)	−0.74	0.460
D1 (positive vs. neutral)	21.91 (8.94)	2.45	0.015	22.07 (8.93)	2.47	0.014	21.99 (8.94)	2.46	0.014
D2 (negative vs. neutral)	30.84 (10.99)	2.81	0.006	30.88 (10.94)	2.82	0.006	30.71 (10.99)	2.79	0.006
Anxiety	1.75 (4.28)	0.41	0.684	1.09 (3.03)	0.36	0.720	-	-	-
Anxiety × D1	−0.11 (1.47)	−0.08	0.940	1.97 (1.06)	1.86	0.064	-	-	-
Anxiety × D2	1.79 (1.81)	0.99	0.327	2.35 (1.3)	1.81	0.073	-	-	-
Depression	−1.05 (4.76)	−0.22	0.826	-	-	-	0.33 (3.36)	0.10	0.923
Depression × D1	3.32 (1.63)	2.04	0.042	-	-	-	3.24 (1.17)	2.76	0.006
Depression × D2	0.89 (2.01)	0.44	0.659	-	-	-	2.27 (1.44)	1.57	0.120

Note. D1 represents the contrast between conditions with positive and neutral stimuli; D2 represents the contrast between conditions with negative and neutral stimuli. SE: standard error.

## Data Availability

Data are available from the authors upon reasonable request.
